# Deductive Stability Proofs for Ordinary Differential Equations

**DOI:** 10.1007/978-3-030-72013-1_10

**Published:** 2021-02-26

**Authors:** Yong Kiam Tan, André Platzer

**Affiliations:** 8 Eindhoven University of Technology, Eindhoven, The Netherlands; 9 Aalborg University, Aalborg East, Denmark; Computer Science Department, Carnegie Mellon University, Pittsburgh, USA

**Keywords:** differential equations, stability, differential dynamic logic

## Abstract

Stability is required for real world controlled systems as it ensures that those systems can tolerate small, real world perturbations around their desired operating states. This paper shows how stability for continuous systems modeled by ordinary differential equations (ODEs) can be formally verified in differential dynamic logic (dL). The key insight is to specify ODE stability by suitably nesting the dynamic modalities of dL with first-order logic quantifiers. Elucidating the logical structure of stability properties in this way has three key benefits: *i)* it provides a flexible means of formally specifying various stability properties of interest, *ii)* it yields rigorous proofs of those stability properties from dL’s axioms with dL’s ODE safety and liveness proof principles, and *iii)* it enables formal analysis of the relationships between various stability properties which, in turn, inform proofs of those properties. These benefits are put into practice through an implementation of stability proofs for several examples in KeYmaera X, a hybrid systems theorem prover based on dL.

### 1 Introduction

The study of stability has its roots in efforts to understand mechanical systems, particularly those arising in celestial mechanics [15,19,30]. Today, it is an important part of numerous applications in dynamical systems [34] and control theory [14,18]. This paper studies proofs of stability for continuous dynamical systems described by *ordinary differential equations* (ODEs), such as those used to model feedback control systems [14,18]. For such systems, ODE stability is a key correctness requirement [2] that deserves fully rigorous proofs *alongside* other key properties such as safety and liveness of those ODEs [28,36]. Despite this, formal stability verification has received less attention compared to proofs of safety and liveness, e.g., through reachability or deductive techniques [8].

Stability for a continuous system (or ODEs) requires that *i)* its system state always stays close to some desired operating state(s) when initially slightly perturbed from those operating state(s), and *ii)* those perturbations are eventually dissipated so the system returns to a desired operating state. These properties

⋆ This research was sponsored by the AFOSR under grant number FA9550-16-1-0288. The first author was supported by A*STAR, Singapore.

are especially crucial for engineered systems because they must be robust to real world perturbations deviating from idealized system models. Simple pendulums provide canonical examples of stability phenomena: they are always observed to settle in the rest position of Fig. 1 (bottom) after some time regardless of how they are initially released. In contrast, the inverted pendulum in Fig. 1 (top) is *theoretically* also at a resting position but can only be observed transiently in practice because the slightest real world perturbation will cause the pendulum to fall due to gravity. Stability explains these observations—the resting position is (asymptotically) stable while the inverted position is unstable and requires active control to ensure its stability. Proofs of safety and liveness properties are still required for the inverted pendulum under control, e.g., its controller must never generate unsafe amounts of torque and the pendulum must eventually reach the inverted position. The *triumvirate* of safety, liveness, and stability is required for holistic correctness of the inverted pendulum controller.

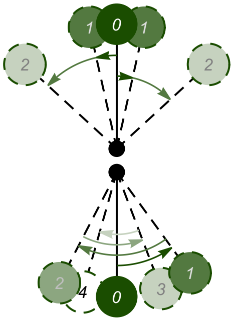

**Fig. 1.** A pendulum (in green) hung by a rigid rod from a pivot (in black) perturbed from its resting state (bottom) and from its inverted, upright position (top). Perturbed states (with dashed boundaries) are faded out to show the progression of time.

The classical way of distinguishing the aforementioned stability situations is by designing a *Lyapunov function* [19], i.e., an energy-like auxiliary measure satisfying certain *arithmetical conditions* [14,18,31] which implies that the auxiliary energy decreases along system trajectories towards local minima at the stable resting state(s), see Fig. 2. Prior approaches [1,12,17,21,33] have emphasized the need to formally verify those arithmetical conditions in order to guarantee that a conjectured Lyapunov function correctly implies stability for a given system.

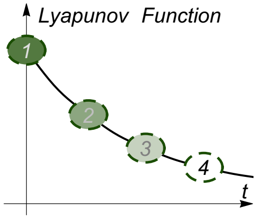

**Fig. 2.** A Lyapunov function that decreases along the pendulum trajectory shown in Fig. 1 (bottom).

This paper shows how deductive proofs of ODE stability can be carried out in differential dynamic logic (dL) [25,26,27], a logic for *deductive verification* of hybrid systems.[1] The key insight is that stability properties can be specified by suitably nesting the dynamic modalities of dL with quantifiers of first-order logic. The resulting specifications are amenable to rigorous proof by combining dL's ODE safety [28] and liveness [36] proof principles with real arithmetic and first-order quantifier reasoning. This makes it possible to *syntactically derive* stability for a given system from the small set of dL axioms which, in turn, enables trustworthy stability proofs in the KeYmaera X theorem prover for hybrid systems [11,26]. Notably, this approach directly verifies *stability specifications*, which

[1] Hybrid systems are mathematical models describing discrete and continuous dynamics, and interactions thereof. This paper's formal understanding of ODE stability is crucial for subsequent investigation of hybrid systems stability [5,13,20].

goes beyond verifying arithmetic that imply those specifications [1,12,17,21,33]. This is crucial for advanced stability notions because those variations generally require subtle twists to the required arithmetical conditions on their Lyapunov functions [14]; proofs of stability specifications alleviate the onus on system designers to correctly pick and check the appropriate conditions for their applications. Section 3 shows how various stability properties for ODE equilibria can be formally specified and proved in dL with Lyapunov function techniques. Section 4 generalizes those stability specifications, yielding unambiguous formal specifications of advanced stability properties from the literature [14,18], along with their derived proof rules. These specifications also provide rigorous insights into the logical relationship between various stability notions, which are used to inform their respective proofs. Section 5 illustrates the practicality of this paper's dL approach through several stability case studies formalized in KeYmaera X.

All omitted definitions and proofs are available in the supplement [35].

### 2 Background: Differential Dynamic Logic

This section briefly recalls the syntax and semantics of dL, focusing on its continuous fragment which has a complete axiomatization for ODE invariants [28]. Full presentations of dL, including its discrete fragment, are elsewhere [26,27].

**Syntax and Semantics.** The grammar of dL terms is as follows, where $x \in \mathcal{V}$ is a variable and $c \in \mathbb{Q}$ is a rational constant. These terms are polynomials over $\mathcal{V}$ (extensions with Noetherian functions [28] such as $\exp, \sin, \cos$ are possible):

$$p, q \ ::= \ x \mid c \mid p + q \mid p \cdot q$$

The grammar of dL formulas is as follows, where $\sim \in \{=, \neq, \geq, >, \leq, <\}$ is a comparison operator and $\alpha$ is a hybrid program:

$$\phi, \psi \ ::= p \sim q \mid \phi \land \psi \mid \phi \lor \psi \mid \neg\phi \mid \forall v\, \phi \mid \exists v\, \phi \mid [\alpha]\phi \mid \langle\alpha\rangle\phi$$

This grammar features atomic comparisons ($p \sim q$), propositional connectives ($\neg$, $\land$, $\lor$), first-order quantifiers over the reals ($\forall$, $\exists$), and the box ($[\alpha]\phi$) and diamond ($\langle\alpha\rangle\phi$) modality formulas which express that all or some runs of hybrid program $\alpha$ satisfy $\phi$, respectively. The modalities $[\cdot], \langle\cdot\rangle$ can be freely nested with first-order and modal connectives, which is crucial for the specification of stability properties in Sections 3 and 4. Formulas not containing the modalities are formulas of first-order real arithmetic and are written as $P, Q, R$.

This paper focuses on the *continuous* fragment of hybrid programs $\alpha \equiv x' = f(x) \,\&\, Q$, where $x' = f(x)$ is an $n$-dimensional system of ordinary differential equations (ODEs), $x_1'{=}f_1(x), \ldots, x_n'{=}f_n(x)$, over variables $x = (x_1, \ldots, x_n)$, the LHS $x_i'$ is the time derivative of $x_i$ and the RHS $f_i(x)$ is a polynomial over variables $x$. The evolution domain constraint $Q$ specifies the set of states in which the ODE is allowed to evolve continuously. When $Q$ is the formula *true*, the ODE is also written as $x' = f(x)$. For $n$-dimensional vectors $x, y$, the dot product is $x \cdot y \overset{\text{def}}{=} \sum_{i=1}^{n} x_i y_i$ and $\|x\|^2 \overset{\text{def}}{=} \sum_{i=1}^{n} x_i^2$ denotes the squared Euclidean norm. Variables $z \in \mathcal{V} \setminus \{x\}$ not occurring on the LHS of ODE $x' = f(x)$ are *parameters* that remain constant along ODE solutions. The following parametric ODE model of a simple pendulum is used as a running example.

*Example 1 (Pendulum model).* The ODE $\alpha_p \equiv \theta' = \omega,\ \omega' = -\frac{g}{L}\sin(\theta) - b\omega$ models a pendulum (illustrated below) suspended from a pivot by a rod of length $L$, where $\theta$ is the angle of displacement, $\omega$ is the angular velocity of the pendulum, and $g > 0$ is the gravitational constant. Parameter $a = \frac{g}{L}$ is a positive scaling constant and parameter $b \geq 0$ is the coefficient of friction for angular velocity. The symbolic parameters $a, b$ make analysis of $\alpha_p$ apply to a range of concrete values, e.g., pendulums that are suspended by a long rod (with large $L$) are modeled by small positive values of $a$, while frictionless pendulums have $b = 0$.

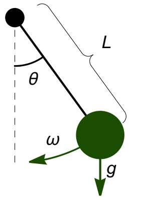

A simplification of $\alpha_p$ is used because stability analyses often concern the behavior of the pendulum near its resting (or inverted) state where $\theta = 0$. For such nearby states with $\theta \approx 0$, the small angle approximation $\sin(\theta) \approx \theta$ yields a linear ODE:[2]

$$\alpha_l \equiv \theta' = \omega,\ \omega' = -a\theta - b\omega \tag{1}$$

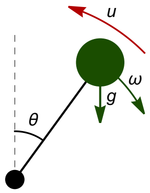

An *inverted* pendulum is modeled by a similar ODE (illustrated on the right) under a change of coordinates. Such a pendulum requires an external torque input $u(\theta, \omega)$ to maintain its stability; $u(\theta, \omega)$ is determined and proved correct in Section 5.

$$\alpha_i \equiv \theta' = \omega,\ \omega' = a\theta - b\omega - u(\theta, \omega) \tag{2}$$

States $\nu : \mathcal{V} \to \mathbb{R}$ assign real values to each variable in $\mathcal{V}$; the set of all states is $\mathbb{S}$. The semantics of dL formula $\phi$ is the set of states $[\![\phi]\!] \subseteq \mathbb{S}$ in which $\phi$ is true [26,27], where the semantics of first-order logical connectives are defined as usual, e.g., $[\![\phi \land \psi]\!] = [\![\phi]\!] \cap [\![\psi]\!]$. For ODEs, the semantics of the modal operators is as follows.[3] Let $\nu \in \mathbb{S}$ and $\boldsymbol{\varphi} : [0, T) \to \mathbb{S}$ for some $0 < T \leq \infty$, be the unique, right-maximal solution [6] to ODE $x' = f(x)$ with initial value $\boldsymbol{\varphi}(0) = \nu$:

$$\nu \in [\![[x' = f(x) \,\&\, Q]\phi]\!] \text{ iff for all } 0 \leq \tau < T \text{ where } \boldsymbol{\varphi}(\zeta) \in [\![Q]\!] \text{ for all } 0 \leq \zeta \leq \tau\text{:}$$
$$\boldsymbol{\varphi}(\tau) \in [\![\phi]\!]$$
$$\nu \in [\![\langle x' = f(x) \,\&\, Q\rangle\phi]\!] \text{ iff there exists } 0 \leq \tau < T \text{ such that:}$$
$$\boldsymbol{\varphi}(\tau) \in [\![\phi]\!] \text{ and } \boldsymbol{\varphi}(\zeta) \in [\![Q]\!] \text{ for all } 0 \leq \zeta \leq \tau$$

For a formula $P$ the $\varepsilon$-neighborhood of $P$ with respect to $x$ is defined as $\mathcal{U}_\varepsilon(P) \overset{\text{def}}{\equiv} \exists y \left(\|x - y\|^2 < \varepsilon^2 \land P(y)\right)$, where the existentially quantified variables $y$ are fresh in $P$. The neighborhood formula $\mathcal{U}_\varepsilon(P)$ characterizes the set of states within distance $\varepsilon$ from $P$, with respect to the dynamically evolving variables $x$.

[2] This linearization is justified by the Hartman-Grobman theorem [6]. A nonlinear polynomial approximation, such as $\sin(\theta) \approx \theta - \frac{\theta^3}{6}$, can also be used.

[3] The semantics of dL formulas is defined compositionally elsewhere [26,27].

This is useful for syntactically expressing small $\varepsilon$ perturbations in the stability definitions of Sections 3 and 4. For formulas $P$ of first-order real arithmetic, the $\varepsilon$-neighborhood, $\mathcal{U}_\varepsilon(P)$, can be equivalently expressed in quantifier-free form by quantifier elimination [4]. For example, $\mathcal{U}_\varepsilon(x = 0)$ is equivalent to the formula $\|x\|^2 < \varepsilon^2$. Formulas $\overline{P}$ and $\partial P$ are the syntactically definable topological closure and boundary of the set characterized by $P$, respectively [4].

**Proof Calculus.** All derivations and proof rules are presented in a classical sequent calculus. The semantics of *sequent* $\Gamma \vdash \phi$ is equivalent to the formula $(\bigwedge_{\psi\in\Gamma} \psi) \rightarrow \phi$. A sequent is valid iff its corresponding formula is valid. Completed branches in a sequent proof are marked with $*$. Assumptions $\psi \in \Gamma$ that have only ODE parameters as free variables remain true along ODE evolutions and are soundly kept across ODE deduction steps [26,27]. First-order real arithmetic is decidable [4] so we assume such a decision procedure and label proof steps with $\mathbb{R}$ when they follow from real arithmetic. Axioms and proof rules are *derivable* iff they can be deduced from sound dL axioms and proof rules [26,27].

Formula $I$ is an *invariant* of the ODE $x' = f(x) \,\&\, Q$ iff the formula $I \rightarrow [x' = f(x) \,\&\, Q]I$ is valid. The dL proof calculus is *complete* for ODE invariants [28], i.e., any true ODE invariant expressible in first-order real arithmetic can be proved in the calculus. The calculus also supports refinement reasoning [36] for proving ODE liveness properties $P \rightarrow \langle x' = f(x) \,\&\, Q\rangle R$, which says that the goal $R$ is reached along the ODE $x' = f(x) \,\&\, Q$ from precondition $P$.

An important syntactic tool for reasoning with ODE $x' = f(x)$ is the *Lie derivative* of term $p$ defined as $\dot{p} \stackrel{\text{def}}{=} \sum_{x_i\in x} \frac{\partial p}{\partial x_i} f_i(x)$, whose semantic value is equal to the time derivative of the value of $p$ along solutions $\boldsymbol{\varphi}$ of the ODE [26,28]. They are provably definable in dL using syntactic differentials [26].

### 3 Asymptotic Stability of an Equilibrium Point

This section presents Lyapunov's classical notion of asymptotic stability [19] and its formal specification in dL. This formalization enables the derivation of dL stability proof rules with *Lyapunov functions* [14,18,19,31]. Several related stability concepts are formalized in dL, along with their relationships and rules.

#### 3.1 Mathematical Preliminaries

An *equilibrium point* of ODE $x' = f(x)$ is a point $x_0 \in \mathbb{R}^n$ where $f(x_0) = 0$, so a system that starts at $x_0$ stays at $x_0$ along its continuous evolution. Such points are often interesting in real-world systems, e.g., the equilibrium point $\theta = 0, \omega = 0$ for $\alpha_l$ from (1) is the resting state of a pendulum. For a controlled system, equilibrium points often correspond to desired steady system states where no further continuous control input (modeled as part of $f(x)$) is required [18].

For brevity, assume the origin $0 \in \mathbb{R}^n$ is an equilibrium point of interest. Any other equilibrium point(s) of interest $x_0 \in \mathbb{R}^n$ can be translated to the origin with the change of coordinates $x \mapsto x - x_0$ for the ODE (see supplement [35]).

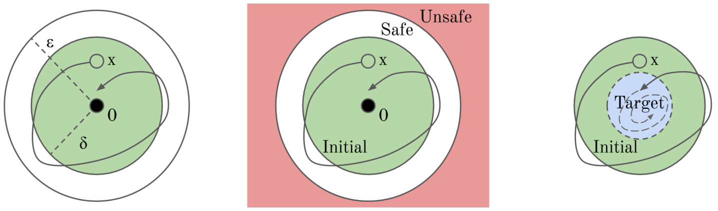

**Fig. 3.** Solutions from points in the $\delta$ ball around the origin, like the green initial point $x$, remain within the $\varepsilon$ ball around the origin $0 \in \mathbb{R}^n$ (black dot) and asymptotically approach the origin. The latter two plots illustrate how asymptotic stability for an ODE can be broken down into a pair of (quantified) ODE safety and liveness properties.

The following definition of asymptotic stability is standard [14,18,31].[4]

**Definition 2 (Asymptotic stability [14,18,31]).** *The origin* $0 \in \mathbb{R}^n$ *of ODE* $x' = f(x)$ *is*

- ***stable*** *if, for all* $\varepsilon > 0$, *there exists* $\delta > 0$ *such that for all initial states* $x = x(0)$ *with* $\|x\| < \delta$, *the right-maximal ODE solution* $x(t) : [0, T) \to \mathbb{R}^n$ *satisfies* $\|x(t)\| < \varepsilon$ *for all times* $0 \leq t < T$,
- ***attractive*** *if there exists* $\delta > 0$ *such that for all* $x = x(0)$ *with* $\|x\| < \delta$, *the right-maximal ODE solution* $x(t) : [0, T) \to \mathbb{R}^n$ *satisfies* $\lim_{t \to T} x(t) = 0$,
- ***asymptotically stable*** *if it is stable and attractive.*

These definitions can be understood using the resting state of the pendulum from Fig. 1 (bottom) which is asymptotically stable. When the pendulum is given a light push from its bottom resting state (formally, $\|x\| < \delta$), it gently oscillates near that resting state (formally, $\|x(t)\| < \varepsilon$). In the presence of friction, these oscillations eventually dissipate so the pendulum asymptotically returns to its resting state (formally, $\lim_{t \to T} x(t) = 0$). This behavior is *local*, i.e., for any given $\varepsilon > 0$, there *exists* a sufficiently small $\delta > 0$ perturbation of the initial state that results in gentle oscillations with $\|x(t)\| < \varepsilon$, see Fig. 3 (left). A strong push, e.g., with $\delta > \varepsilon$, could instead cause the pendulum to spin around on its pivot.

*Remark 3.* Stability and attractivity *do not* imply each other [31, Chapter I.2.7]. However, if the origin is stable, attractivity can be defined in a simpler way. This is proved in dL, after characterizing stability and attractivity syntactically.

#### 3.2 Formal Specification

The formal specification of asymptotic stability in dL combines *i)* the dynamic modalities of dL, which are used to quantify over the dynamics of the ODE, and *ii)* the first-order logic quantifiers, which are used to express combinations of (topologically) local and asymptotic properties of those dynamics.

[4] Some definitions require, or implicitly assume, right-maximal solutions $x(t)$ to be global, i.e., with $T = \infty$, see [18, Definition 4.1] and associated discussion. The definitions presented here are better suited for subsequent generalizations.

**Lemma 4 (Asymptotic stability in dL).** *The origin of ODE $x' = f(x)$ is, respectively, i)* ***stable****, ii)* ***attractive****, and iii)* ***asymptotically stable*** *iff the* dL *formulas i)* $\text{Stab}(x' = f(x))$*, ii)* $\text{Attr}(x' = f(x))$*, and iii)* $\text{AStab}(x' = f(x))$ *respectively are valid. Variables $\varepsilon, \delta$ are fresh, i.e., not in $x, f(x)$.*

$$\text{Stab}(x' = f(x)) \equiv \forall \varepsilon{>}0\, \exists \delta{>}0\, \forall x \left(\mathcal{U}_\delta(x = 0) \rightarrow [x' = f(x)]\, \mathcal{U}_\varepsilon(x = 0)\right)$$
$$\text{Attr}(x' = f(x)) \equiv \exists \delta{>}0\, \forall x \left(\mathcal{U}_\delta(x = 0) \rightarrow \text{Asym}(x' = f(x), x = 0)\right)$$
$$\text{AStab}(x' = f(x)) \equiv \text{Stab}(x' = f(x)) \wedge \text{Attr}(x' = f(x))$$

*Formula $\text{Asym}(x' = f(x), P) \equiv \forall \varepsilon{>}0\, \langle x' = f(x)\rangle [x' = f(x)]\, \mathcal{U}_\varepsilon(P)$ characterizes the set of states that asymptotically approach $P$ along ODE solutions.*

Formula $\text{Stab}(x' = f(x))$ is a syntactic dL rendering of the corresponding quantifiers from Def. 2. The safety property $\mathcal{U}_\delta(x = 0) \rightarrow [x' = f(x)]\, \mathcal{U}_\varepsilon(x = 0)$ expresses that solutions starting from the $\delta$-neighborhood of the origin always (for all times) stay safely in the $\varepsilon$-neighborhood, as visualized in Fig. 3 (middle).

Formula $\text{Attr}(x' = f(x))$ uses the subformula $\text{Asym}(x' = f(x), x = 0)$ which characterizes the limit in Def. 2. Recall $\lim_{t \to T} x(t) = 0$ iff for all $\varepsilon > 0$ there exists a time $\tau$ with $0 \le \tau < T$ such that for all times $t$ with $\tau \le t < T$, the solution satisfies $\|x(t)\| < \varepsilon$, i.e., the limit requires for all distances $\varepsilon > 0$, the ODE solution will *eventually always* be within distance $\varepsilon$ of the origin, as visualized in Fig. 3 (right). This limit is characterized using nested $\langle\cdot\rangle[\cdot]$ modalities, together with first-order quantification according to Def. 2. More generally, formula $\text{Asym}(x' = f(x), P)$ characterizes the set of initial states where the right-maximal ODE solution asymptotically approaches $P$; this set is known as the *region of attraction* of $P$ [18]. Thus, attractivity requires that the region of attraction of the origin contains an open neighborhood $\mathcal{U}_\delta(x = 0)$ of the origin.

From Lemma 4, proving validity of the formula $\text{AStab}(x' = f(x))$ yields a rigorous proof of asymptotic stability for $x' = f(x)$. However, if the origin is stable, then attractivity can be provably simplified with the following corollary.

**Corollary 5 (Stable attractivity).** *The following axiom is derivable in* dL*.*

SAttr $\text{Stab}(x' = f(x)) \rightarrow \left(\text{Asym}(x' = f(x), x{=}0) \leftrightarrow \forall \varepsilon{>}0\, \langle x' = f(x)\rangle\, \mathcal{U}_\varepsilon(x{=}0)\right)$

Corollary 5 simplifies the syntactic characterization of the region of attraction for stable equilibria from a nested $\langle\cdot\rangle[\cdot]$ formula to a $\langle\cdot\rangle$ formula, which is then directly amenable to ODE liveness reasoning [36]. This corollary is used to simplify proofs of asymptotic stability, as explained next.

#### 3.3 Lyapunov Functions

*Lyapunov functions* are the standard tool for showing stability of general, nonlinear ODEs [14,18,31] and finding suitable Lyapunov functions is an important problem in its own right [1,9,12,17,21,23,24,33,37]. This section shows how a candidate Lyapunov function, once found, can be used to rigorously prove stability. The following proof rules derive Lyapunov stability arguments [14,18,31] syntactically in dL.

**Lemma 6 (Lyapunov functions).** *The following Lyapunov function proof rules are derivable in* dL.

$$\text{Lyap}_{\geq}\ \frac{\vdash f(0)=0 \land v(0)=0 \quad \vdash \exists\gamma{>}0\,\forall x\,\big(0{<}\|x\|^2{\leq}\gamma^2 \to v>0 \land \dot{v} \leq 0\big)}{\vdash \text{Stab}(x'=f(x))}$$

$$\text{Lyap}_{>}\ \frac{\vdash f(0)=0 \land v(0)=0 \quad \vdash \exists\gamma{>}0\,\forall x\,\big(0{<}\|x\|^2{\leq}\gamma^2 \to v>0 \land \dot{v} < 0\big)}{\vdash \text{AStab}(x'=f(x))}$$

Rules $\text{Lyap}_{\geq}$, $\text{Lyap}_{>}$ use the Lyapunov function $v$ as an auxiliary, energy-like function near the origin which is positive and has non-positive (resp. negative $\text{Lyap}_{>}$) derivative $\dot{v}$. This guarantees that $v$ is non-increasing (resp. decreasing) along ODE solutions near the origin, see Fig. 2. The right premise of both rules use $\exists\gamma{>}0\,\forall x\,\big(0{<}\|x\|^2{\leq}\gamma^2 \to \cdots\big)$ to require that the Lyapunov function conditions are true in a $\gamma$-neighborhood of the origin. The subtle difference in sign condition for $\dot{v}$ between rules $\text{Lyap}_{\geq}$, $\text{Lyap}_{>}$ is illustrated for the pendulum.

*Example 7 (Pendulum asymptotic stability).* For ODE $\alpha_l$ from (1), a suitable Lyapunov function for proving its stability [18] is $v = a\frac{\theta^2}{2} + \frac{(b\theta+\omega)^2+\omega^2}{4}$, where the Lie derivative of $v$ along $\alpha_l$ is $\dot{v} = -\frac{b}{2}(a\theta^2+\omega^2)$. Stability[5] is formally proved in dL for *any* parameter values $a > 0, b \geq 0$ using rule $\text{Lyap}_{\geq}$ because both of its resulting arithmetical premises are provable by $\mathbb{R}$. The full dL derivation, also used in KeYmaera X (Section 5), is shown in the proof of Lemma 6 [35].

When $b > 0$, i.e., friction is non-negligible, an identical derivation with $\text{Lyap}_{>}$ instead of $\text{Lyap}_{\geq}$ proves asymptotic stability because $-\frac{b}{2}(a\theta^2 + \omega^2)$ is negative except at the origin. Indeed, displacements to the pendulum's resting state can only be dissipated in the presence of friction, not when $b = 0$.

#### 3.4 Asymptotic Stability Variations

Asymptotic stability is a strong guarantee about the local behavior of ODE solutions near equilibrium points of interest. In certain applications, stronger stability guarantees may be needed for those equilibria [18]. This section examines two standard stability variations, shows how they can be proved in dL, and formally analyzes their logical relationship with asymptotic stability.

**Exponential stability** As the name suggests, the first stability variation, exponential stability, guarantees an exponential rate of convergence towards the equilibrium point from an initial displacement. This is useful, e.g., for bounding the time spent by a perturbed system far away from its desired operating state.

**Definition 8 (Exponential stability [14,18,31]).** *The origin* $0 \in \mathbb{R}^n$ *of ODE* $x' = f(x)$ *is* ***exponentially stable*** *if there are positive constants* $\alpha, \beta, \delta > 0$ *such that for all initial states* $x = x(0)$ *with* $\|x\| < \delta$*, the right-maximal ODE solution* $x(t) : [0, T) \to \mathbb{R}^n$ *satisfies* $\|x(t)\| \leq \alpha\|x(0)\| \exp(-\beta t)$ *for all times* $0 \leq t < T$.

[5] For the trigonometric pendulum ODE $\alpha_p$ from Example 1, the Lyapunov function $v = a(1-\cos(\theta)) + \frac{(b\theta+\omega)^2+\omega^2}{4}$ with Lie derivative $\dot{v} = -\frac{b}{2}(a\theta\sin(\theta) + \omega^2)$ proves its stability [18] but requires arithmetic reasoning over trigonometric functions.

Exponential stability bounds the norm of solutions to ODE $x' = f(x)$ near the origin by a decaying exponential. It is specified in dL as follows.

**Lemma 9 (Exponential stability in dL).** *The origin of ODE $x' = f(x)$ is **exponentially stable** iff the following* dL *formula is valid. Variables $\alpha, \beta, \delta, y$ are fresh, i.e., not in $x, f(x)$.*

$$\begin{aligned}\text{EStab}(x' = f(x)) \equiv \exists\alpha{>}0\,\exists\beta{>}0\,\exists\delta{>}0\,\forall x\, \big(&\mathcal{U}_\delta(x = 0) \rightarrow \\ &[y := \alpha^2\|x\|^2; x' = f(x), y' = -2\beta y]\, \|x\|^2 \le y\big)\end{aligned}$$

*The discrete assignment $y := \alpha^2\|x\|^2$ sets the value of variable $y$ to that of $\alpha^2\|x\|^2$ and ; denotes sequential composition of hybrid programs [26,27].*

Formula $\text{EStab}(x' = f(x))$ uses a fresh variable $y$ with ODE $y' = -2\beta y$ and initialized to $\alpha^2\|x\|^2$ so that $y$ *differentially axiomatizes* [28] the (squared) decaying exponential function $\alpha^2\|x(0)\|^2 \exp(-2\beta t)$ along ODE solutions. Such an implicit (polynomial) characterization of exponential decay allows syntactic proof steps to use decidable real arithmetic reasoning.

**Lemma 10 (Lyapunov function for exponential stability).** *The following Lyapunov function proof rule for exponential stability is derivable in* dL*, where $k_1, k_2, k_3 \in \mathbb{Q}$ are positive constants.*

$$\text{Lyap}_\text{E}\quad \frac{\vdash \exists\gamma{>}0\,\forall x\,\big(\|x\|^2{\le}\gamma^2 \rightarrow k_1^2\|x\|^2 \le v \le k_2^2\|x\|^2 \wedge \dot{v} \le -2k_3 v\big)}{\vdash \text{EStab}(x' = f(x))}$$

Rule $\text{Lyap}_\text{E}$ enables proofs of exponential stability in dL. In fact, the proof of Lemma 10 (see supplement [35]) yields concrete, *quantitative* bounds, where $\text{EStab}(x' = f(x))$ is explicitly witnessed with scaling constant $\alpha = \frac{k_2}{k_1}$ and decay rate $\beta = k_3$. These can be used to calculate time bounds when the system state will return sufficiently close to the origin. Similarly, the disturbance $\delta$ in $\text{EStab}(x' = f(x))$ is quantitatively witnessed by $\frac{k_1}{k_2}\gamma$ for any $\gamma$ witnessing validity of the premise of rule $\text{Lyap}_\text{E}$. This yields a provable estimate of the region around the origin where exponential stability holds; this latter estimate is explored next.

**Region of attraction** Formulas $\text{Attr}(x' = f(x))$ and $\text{EStab}(x' = f(x))$ both feature a subformula of the form $\exists\delta > 0\,\forall x\,(\mathcal{U}_\delta(x = 0) \rightarrow \cdots)$ which expresses that attractivity (or exponential stability) is locally true in *some* $\delta$ neighborhood of the origin. In many applications, it is useful to find and rigorously prove that a given set is attractive or exponentially stable with respect to the origin [18, Chapter 8.2]. The second stability variation yields *provable* subsets of the region of attraction, including the special case where it is the entire state space. This is formalized using the following variants of $\text{Attr}(x' = f(x))$ and $\text{EStab}(x' = f(x))$ within a region given by a formula $P$.

$$\begin{aligned}\text{Attr}^\text{P}(x' = f(x), P) &\equiv \forall x\,\big(P \rightarrow \text{Asym}(x' = f(x), x = 0)\big)\\ \text{EStab}^\text{P}(x' = f(x), P) &\equiv \exists\alpha{>}0\,\exists\beta{>}0\,\forall x\,\big(P \rightarrow \\ &\qquad [y := \alpha^2\|x\|^2; x' = f(x), y' = -2\beta y]\, \|x\|^2 \le y\big)\end{aligned}$$

The formula $\text{Attr}^{\text{P}}(x' = f(x), P)$ is valid iff the set characterized by $P$ is a subset of the origin's region of attraction [18]. For example, $\text{Attr}(x' = f(x))$ is $\exists \delta > 0\ \text{Attr}^{\text{P}}(x' = f(x), \mathcal{U}_\delta(x = 0))$. This generalization is useful for formalizing stronger notions of stability in dL, such as the following *global* stability notions [14,18]. For brevity, dL specifications of the stability properties (in **bold**) are given below with mathematical definitions deferred to the supplement [35].

**Lemma 11 (Global stability in dL).** *The origin of ODE $x' = f(x)$ is **globally asymptotically stable** iff the* dL *formula* $\text{Stab}(x' = f(x)) \land \text{Attr}^{\text{P}}(x' = f(x), \mathit{true})$ *is valid. The origin is **globally exponentially stable** iff the* dL *formula* $\text{EStab}^{\text{P}}(x' = f(x), \mathit{true})$ *is valid.*

Global stability ensures that *all* perturbations to the system state are eventually dissipated. Their proof rules are similar to $\text{Lyap}_>$ and $\text{Lyap}_{\text{E}}$ respectively.

**Lemma 12 (Lyapunov function for global stability).** *The following Lyapunov function proof rules for global asymptotic and exponential stability are derivable in* dL*. In rule $\text{Lyap}^{G}_{E}$, $k_1, k_2, k_3 \in \mathbb{Q}$ are positive constants.*

$$\text{Lyap}^{\text{G}}_{>}\ \frac{\vdash f(0){=}0 \land v(0){=}0 \quad x{\neq}0 \vdash v{>}0 \land \dot{v}{<}0 \quad \vdash \forall b\, \exists \gamma{>}0\, \forall x\, \big(v{\leq}b{\rightarrow}\mathcal{U}_\gamma(x{=}0)\big)}{\vdash \text{Stab}(x' = f(x)) \land \text{Attr}^{\text{P}}(x' = f(x), \mathit{true})}$$

$$\text{Lyap}^{\text{G}}_{\text{E}}\ \frac{\vdash k_1^2\|x\|^2 \leq v \leq k_2^2\|x\|^2 \land \dot{v} \leq -2k_3 v}{\vdash \text{EStab}^{\text{P}}(x' = f(x), \mathit{true})}$$

*Example 13 (Pendulum global exponential stability).* For simplicity, instantiate Example 7 with parameters $a = 1, b = 1$. The Lyapunov function then simplifies to $v = \frac{\theta^2}{2} + \frac{(\theta+\omega)^2+\omega^2}{4}$ with Lie derivative $\dot{v} = -\frac{(\theta^2+\omega^2)}{2}$, which satisfies the real arithmetic inequalities $\frac{\theta^2+\omega^2}{4} \leq v \leq \theta^2 + \omega^2$ and $\dot{v} \leq -\frac{1}{2}v$. Thus, rule $\text{Lyap}^{\text{G}}_{\text{E}}$ proves global exponential stability of $\alpha_l$ with $k_1 = \frac{1}{2}$, $k_2 = 1$, and $k_3 = \frac{1}{4}$. An important caveat is that Example 7 used a local small angle approximation, so this global phenomenon does *not* hold for a real world pendulum (nor for $\alpha_p$).

**Logical relationships** With the proliferation of stability variations just introduced, it is useful to take stock of their logical relationships. An important example of such a relationship is shown in the following corollary.

**Corollary 14 (Exponential stability implies asymptotic stability).** *The following axioms are derivable in* dL*.*

EStabStab $\text{EStab}(x' = f(x)) \rightarrow \text{Stab}(x' = f(x))$

EStabAttr $\text{EStab}^{\text{P}}(x' = f(x), P) \rightarrow \text{Attr}^{\text{P}}(x' = f(x), P)$

Derived axioms EStabStab, EStabAttr show that exponential stability implies asymptotic stability. In proofs, EStabAttr allows the region of attraction to be estimated using the region where solutions are exponentially bounded.

## 4 General Stability

This section provides stability definitions and proof rules that generalize stability for an equilibrium point from Section 3 to the stability of sets. These definitions are useful when the desired stable system state(s) is not modeled by a single equilibrium point, but may instead, e.g., lie on a periodic trajectory [18], a hyperplane, or a continuum of equilibrium points within the state space [14]. The generalized definition is used to formalize two stability notions from the literature [14,18], and to justify their Lyapunov function proof rules.

### 4.1 General Stability and General Attractivity

The following *general stability* formula defines stability in dL with respect to an ODE $x' = f(x)$ and formulas $P, R$. The quantified variables $\varepsilon, \delta$ are assumed to be fresh by bound renaming, i.e., do not appear in $x, f(x), P$ or $R$.

$$\mathrm{Stab}_{\mathrm{R}}^{\mathrm{P}}(x' = f(x), P, R) \equiv \forall \varepsilon{>}0\, \exists \delta{>}0\, \forall x\, \big(\mathcal{U}_\delta(P) \to [x' = f(x)]\, \mathcal{U}_\varepsilon(R)\big)$$

This formula generalizes stability of the origin $\mathrm{Stab}(x' = f(x))$ by adding two logical tuning knobs that can be intuitively understood as follows. The *precondition* $P$ characterizes the initial states from which the system state is expected to be disturbed by some disturbance $\delta$. The *postcondition* $R$ characterizes the set of desired operating states that the system must remain close (within the $\varepsilon$ neighborhood of $R$) after being disturbed from its initial states.

The *general attractivity* formula similarly generalizes $\mathrm{Attr}^{\mathrm{P}}(x' = f(x), P)$ with a postcondition $R$ towards which the ODE solutions from initial states satisfying precondition $P$ are asymptotically attracted.

$$\mathrm{Attr}_{\mathrm{R}}^{\mathrm{P}}(x' = f(x), P, R) \equiv \forall x\, \big(P \to \mathrm{Asym}(x' = f(x), R)\big)$$

**Lemma 15 (General Lyapunov functions).** *The following Lyapunov function proof rule for general stability with two stacked premises is derivable in* dL*.*

$$\text{GLyap}\ \frac{\begin{array}{l}\vdash P \to R\\ \vdash \forall \varepsilon{>}0\, \exists 0{<}\gamma{\leq}\varepsilon\, \exists k \left(\begin{array}{l}\forall x\, (\partial(\mathcal{U}_\gamma(R)) \to v \geq k) \land\\ \exists 0{<}\delta{\leq}\gamma\, \forall x\, (\mathcal{U}_\delta(P) \to R \lor v{<}k) \land\\ \forall x\, \big(R{\lor}v{<}k \to [x' = f(x)\, \&\, \overline{\mathcal{U}_\gamma(R)}](R{\lor}v{<}k)\big)\end{array}\right)\end{array}}{\vdash \mathrm{Stab}_{\mathrm{R}}^{\mathrm{P}}(x' = f(x), P, R)}$$

Rule GLyap proves general stability for precondition $P$ and postcondition $R$. It generalizes the Lyapunov function reasoning underlying rule $\mathrm{Lyap}_{\geq}$ to support arbitrary pre- and postconditions. The conjunct $\forall x\, (\partial(\mathcal{U}_\gamma(R)) \to v \geq k)$ requires $v{\geq}k$ on the boundary of $\mathcal{U}_\gamma(R)$ while the middle conjunct requires $v{<}k$ for some small neighborhood of $P$ excluding $R$. The conjunct $\forall x\, \big(R{\lor}v{<}k \to \cdots\big)$ asserts that $R \lor v < k$ is an invariant of the ODE *within* closed domain $\overline{\mathcal{U}_\gamma(R)}$. When $R$ is a formula of first-order real arithmetic, this invariance question is provably equivalent in dL to a formula of real arithmetic [28], so the premise of rule GLyap is, *in theory*, decidable by $\mathbb{R}$ for a given candidate Lyapunov function $v$. In practice, it is prudent to consider specialized stability notions, for which the premise of rule GLyap can be arithmetically simplified. Proof rules for generalized attractivity are also derivable for specialized instances.

#### 4.2 Specialization

General stability specializes to several stability notions in the literature. For brevity, dL specifications of the stability properties (in **bold**) are given below with mathematical definitions deferred to the supplement [35].

**Set Stability** An important special case is when the desired operating states are exactly the states from which disturbances are expected, i.e., $R \equiv P$. This leads to the notion of **set stability** of the set characterized by $P$ [14,18].

**Lemma 16 (Set Stability in dL).** *For the ODE $x' = f(x)$, the set characterized by formula $P$ is i) **stable**, ii) **attractive**, iii) **asymptotically stable**, and iv) **globally asymptotically stable** iff the following* dL *formulas are valid:*

*i)* $\mathrm{Stab}^{\mathrm{P}}_{\mathrm{R}}(x' = f(x), P, P)$,
*ii)* $\exists \delta{>}0\ \mathrm{Attr}^{\mathrm{P}}_{\mathrm{R}}(x' = f(x), \mathcal{U}_\delta(P), P)$,
*iii)* $\mathrm{Stab}^{\mathrm{P}}_{\mathrm{R}}(x' = f(x), P, P) \land \exists \delta{>}0\ \mathrm{Attr}^{\mathrm{P}}_{\mathrm{R}}(x' = f(x), \mathcal{U}_\delta(P), P)$, *and*
*iv)* $\mathrm{Stab}^{\mathrm{P}}_{\mathrm{R}}(x' = f(x), P, P) \land \mathrm{Attr}^{\mathrm{P}}_{\mathrm{R}}(x' = f(x), \mathit{true}, P)$

The intuition for Lemma 16 is similar to Lemmas 4 and 11, except formula $P$ (instead of the origin) characterizes the set of desirable states. An application of set stability is shown in the following example.

*Example 17 (Tennis racket theorem [3]).* The following system of ODEs models the rotation of a 3D rigid body [6,14], where $x_1, x_2, x_3$ are angular velocities and $I_1 > I_2 > I_3 > 0$ are the principal moments of inertia along the respective axes.

$$\alpha_r \equiv x_1' = \frac{I_2 - I_3}{I_1} x_2 x_3, \quad x_2' = \frac{I_3 - I_1}{I_2} x_3 x_1, \quad x_3' = \frac{I_1 - I_2}{I_3} x_1 x_2$$

When such a rigid object is spun or rotated on each of its axes, a well-known physical curiosity [3] is that the rotation is stable in the first and third axes, whilst additional (unstable) twisting motion is observed for the intermediate axis. Mathematically, a perfect rotation, e.g., around $x_1$, corresponds to a (large) initial value for $x_1$ with no rotation in the other axes, i.e., $x_2 = 0$, $x_3 = 0$. Accordingly the real world observation of stability for rotations about the first principal axis is explained by stability with respect to small perturbations in $x_2, x_3$, as formally specified by formula (3) below. Note that the set characterized by formula $x_2 = 0 \land x_3 = 0$ is the entire $x_1$ axis, not just a single point. Similarly, rotations are stable around the third principal axis iff formula (4) is valid.

$$\mathrm{Stab}^{\mathrm{P}}_{\mathrm{R}}(\alpha_r, x_2 = 0 \land x_3 = 0, x_2 = 0 \land x_3 = 0) \tag{3}$$

$$\mathrm{Stab}^{\mathrm{P}}_{\mathrm{R}}(\alpha_r, x_1 = 0 \land x_2 = 0, x_1 = 0 \land x_2 = 0) \tag{4}$$

The validity of formulas (3) and (4) are proved in Example 20.

The formal specification of set stability yields three provable logical consequences which are important stepping stones for the set stability proof rules.

**Corollary 18 (Set stability properties).** *The following axioms are derivable in* dL. *In axiom SClosure, formula $\overline{P}$ characterizes the topological closure of formula $P$. In axiom SClosed, formula $P$ characterizes a closed set.*

$$\text{SetSAttr}\quad \text{Stab}^{\text{P}}_{\text{R}}(x'=f(x),P,P) \rightarrow \big(\text{Asym}(x'=f(x),P) \leftrightarrow \forall\varepsilon{>}0\,\langle x'=f(x)\rangle\,\mathcal{U}_\varepsilon(P)\big)$$

$$\text{SClosure}\quad \text{Stab}^{\text{P}}_{\text{R}}(x'=f(x),P,P) \leftrightarrow \text{Stab}^{\text{P}}_{\text{R}}(x'=f(x),\overline{P},\overline{P})$$

$$\text{SClosed}\quad \text{Stab}^{\text{P}}_{\text{R}}(x'=f(x),P,P) \rightarrow \forall x\,\big(P \rightarrow [x'=f(x)]P\big)$$

Axiom SetSAttr generalizes SAttr and provides a syntactic simplification of the region of attraction for formula $P$ when $P$ is stable. Axiom SClosure says that stability of $P$ is equivalent to stability of its closure $\overline{P}$, because for any perturbation $\delta > 0$, the neighborhoods $\mathcal{U}_\delta(P)$ and $\mathcal{U}_\delta(\overline{P})$ are provably equivalent in real arithmetic. Axiom SClosed says that for closed formulas $P$, invariance of $P$ is a necessary condition for stability of $P$. Without loss of generality, it suffices to develop proof rules for stability of formulas characterizing closed (using SClosure) and invariant (using SClosed) sets. Indeed, standard definitions of set stability [14,18] usually assume that the set of concern is closed and invariant.

**Lemma 19 (Set stability Lyapunov functions).** *The following Lyapunov function proof rules for set stability are derivable in* dL. *In derived rules $SLyap_{\geq}$ and $SLyap_{>}$, formula $P$ characterizes a compact (i.e., closed and bounded) set. In derived rule $SLyap^*_{\geq}$, the two premises are stacked.*

$$\text{SLyap}_{\geq}\quad \frac{P \vdash [x'=f(x)]P \qquad \neg P \vdash v > 0 \wedge \dot{v} \leq 0 \qquad \partial P \vdash v \leq 0}{\vdash \text{Stab}^{\text{P}}_{\text{R}}(x'=f(x),P,P)}$$

$$\text{SLyap}_{>}\quad \frac{P \vdash [x'=f(x)]P \qquad \neg P \vdash v > 0 \wedge \dot{v} < 0 \qquad \partial P \vdash v \leq 0}{\vdash \text{Stab}^{\text{P}}_{\text{R}}(x'=f(x),P,P) \wedge \exists\delta{>}0\ \text{Attr}^{\text{P}}_{\text{R}}(x'=f(x),\mathcal{U}_\delta(P),P)}$$

$$\text{SLyap}^*_{\geq}\quad \frac{\begin{array}{l} P \vdash [x'=f(x)]P \\ \vdash \forall\varepsilon{>}0\,\exists 0{<}\gamma{\leq}\varepsilon \left(\begin{array}{l}\exists k \left(\begin{array}{l}\forall x\,(\partial(\mathcal{U}_\gamma(P)) \rightarrow v \geq k) \wedge \\ \exists 0{<}\delta{\leq}\gamma\,\forall x\,(\mathcal{U}_\delta(P) \wedge \neg P \rightarrow v < k)\end{array}\right) \wedge \\ \forall x\,(\overline{\mathcal{U}_\gamma(P)} \wedge \neg P \rightarrow \dot{v} \leq 0)\end{array}\right)\end{array}}{\vdash \text{Stab}^{\text{P}}_{\text{R}}(x'=f(x),P,P)}$$

All three proof rules have the necessary premise $P \vdash [x'=f(x)]P$ which says that formula $P$ is an invariant of the ODE $x' = f(x)$. Rules $SLyap_{\geq}$, $SLyap_{>}$ are slight generalizations of Lyapunov function proof rules for set stability [14] and they respectively generalize rules $Lyap_{\geq}$, $Lyap_{>}$ to prove stability for an invariant $P$. Importantly, both rules assume that $P$ characterizes a compact, i.e., closed and bounded set, which simplifies the arithmetical conditions on $v$ in their premises. The rule *without* the boundedness requirement on $P$ suggested in the remark after [18, Definition 8.1], is unsound, see supplement [35].

For asymptotic stability (in rule SLyap$_>$), boundedness also guarantees that perturbed ODE solutions always exist for sufficient duration, which is a fundamental step in the ODE liveness proofs [36]. Rule SLyap$^*_\geq$ is derived from rule GLyap using invariance of $P$ by the first premise; it provides a means of formally proving the set stability properties (3) and (4) from Example 17.

*Example 20 (Stability of rigid body motion).* The proof for (3) uses the Lyapunov function $v = \frac{1}{2}(\frac{I_1-I_2}{I_3}x_2^2 - \frac{I_3-I_1}{I_2}x_3^2)$, whose Lie derivative is $\dot{v} = 0$, and rule SLyap$^*_\geq$ with formula $P \equiv x_2 = 0 \land x_3 = 0$. The proof for (4) is symmetric. For the top premise of rule SLyap$^*_\geq$, formula $P$ is a provable invariant [28] of the ODE $\alpha_r$. The bottom premise, although arithmetically complicated, can be simplified by choosing $\gamma = \varepsilon$ and deciding the resulting formula by $\mathbb{R}$.

Recall that the $x_1$ axis is *not* a compact set so neither of the standard proof rules for set stability SLyap$_\geq$, SLyap$_>$ would be sound for this proof.

**Epsilon-Stability** Motivated by numerical robustness of proofs of stability, Gao et al. [12] define $\varepsilon$-stability for ODEs. The following dL characterization shows how $\varepsilon$-stability can be understood as an instance of general stability.

**Lemma 21 ($\varepsilon$-Stability in dL).** *The origin of ODE $x' = f(x)$ is **$\varepsilon$-stable** for constant $\varepsilon > 0$ iff the* dL *formula* $\mathrm{Stab}^{\mathrm{P}}_{\mathrm{R}}(x' = f(x), x = 0, \mathcal{U}_\varepsilon(x = 0))$ *is valid.*

Unlike set stability, $\varepsilon$-stability is an instance of general stability where the pre- and postconditions differ. In $\varepsilon$-stability, systems are perturbed from the precondition $x = 0$ (the origin), but the postcondition enlarges the set of desired states to a $\varepsilon > 0$ neighborhood of the origin, which is considered indistinguishable from the origin itself [12]. An immediate consequence of Lemma 21 is that rule GLyap can be used to prove $\varepsilon$-stability, as shown in the next section.

### 5 Stability in KeYmaera X

This section puts the dL stability specifications and derivations from the preceding sections into practice through proofs for several case studies in the KeYmaera X theorem prover [11].[6] Examples 7, 13, 17, 20 have also been formalized. The insights from these proofs are discussed after an overview of the case studies.

*Inverted Pendulum.* The stability of the resting state of the pendulum is investigated in Examples 7 and 13. For the inverted pendulum $\alpha_i$ from (2), the controlled torque $u(\theta, \omega)$ must be designed and rigorously proved to ensure *feedback stabilization* [18] of the inverted position. A standard PD (Proportional-Derivative) controller can be used for stabilization, where the control input has the form $u(\theta, \omega) = k_1\theta + k_2\omega$ for tuning parameters $k_1, k_2$. Asymptotic stability of the inverted position is achieved for any control parameter choice where $k_1 > a$ and $k_2 > -b$. The sequent $a > 0, b \geq 0, k_1 > a, k_2 > -b \vdash \mathrm{AStab}(\alpha_i)$ is proved in KeYmaera X using the Lyapunov function $\frac{(k_1-a)\theta^2}{2} + \frac{(((b+k_2)\theta+\omega)^2+\omega^2)}{4}$.

[6] See https://github.com/LS-Lab/KeYmaeraX-projects/blob/master/stability

*Frictional Tennis Racket Theorem.* The stability of a 3D rigid body is investigated for $\alpha_r$ in Examples 17 and 20. The following ODEs model additional frictional forces that oppose the rotational motion in each axis of the rigid body, where $\alpha_1, \alpha_2, \alpha_3 > 0$ are positive coefficients of friction:

$$\alpha_f \equiv x_1' = \frac{I_2 - I_3}{I_1} x_2 x_3 - \alpha_1 x_1,\ x_2' = \frac{I_3 - I_1}{I_2} x_3 x_1 - \alpha_2 x_2,\ x_3' = \frac{I_1 - I_2}{I_3} x_1 x_2 - \alpha_3 x_3$$

In the presence of friction, rotations of the rigid body are globally asymptotically stable in the first and third principal axes, as proved in KeYmaera X.

$$\Gamma \equiv I_1 > I_2, I_2 > I_3, I_3 > 0, \alpha_1 > 0, \alpha_2 > 0, \alpha_3 > 0$$
$$\Gamma \vdash \mathrm{Stab}_{\mathrm{R}}^{\mathrm{P}}(\alpha_f, x_2{=}0 \wedge x_3{=}0, x_2{=}0 \wedge x_3{=}0) \wedge \mathrm{Attr}_{\mathrm{R}}^{\mathrm{P}}(\alpha_f, \mathit{true}, x_2{=}0 \wedge x_3{=}0)$$
$$\Gamma \vdash \mathrm{Stab}_{\mathrm{R}}^{\mathrm{P}}(\alpha_f, x_1{=}0 \wedge x_2{=}0, x_1{=}0 \wedge x_2{=}0) \wedge \mathrm{Attr}_{\mathrm{R}}^{\mathrm{P}}(\alpha_f, \mathit{true}, x_1{=}0 \wedge x_2{=}0)$$

Both asymptotic stability properties are proved using $\mathrm{SLyap}_{\geq}^{*}$ and the liveness property [36] that the kinetic energy $I_1 x_1^2 + I_2 x_2^2 + I_3 x_3^2$ of the system tends to zero over time. The latter property implies that solutions of $\alpha_f$ exist globally and that the values of $x_1, x_2, x_3$ asymptotically tend to zero, which proves global asymptotic stability with the aid of SetSAttr. Even though a proof rule for (global) asymptotic stability of general nonlinear ODEs and unbounded sets is not available (Section 4), this example shows that formalized stability properties can still be proved on a case-by-case basis using dL's ODE reasoning principles.

*Moore-Greitzer Jet Engine [12].* The origin of the ODE modeling a simplified jet engine $\alpha_m \equiv x_1' = -x_2 - \frac{3}{2}x_1^2 - \frac{1}{2}x_1^3,\ x_2' = 3x_1 - x_2$ is $\varepsilon$-stable for $\varepsilon = 10^{-10}$ [12]. The sequent $\varepsilon = 10^{-10} \vdash \mathrm{Stab}_{\mathrm{R}}^{\mathrm{P}}(\alpha_m, x_1^2 + x_2^2 = 0, x_1^2 + x_2^2 < \varepsilon^2)$ is proved in KeYmaera X. The key proof ingredients are an $\varepsilon$-Lyapunov function [12] and manual arithmetic steps, e.g., instantiating existential quantifiers appearing in the specification of $\varepsilon$-stability with appropriate values [12].

*Other Examples [1].* Stability for several ODEs with Lyapunov functions generated by an inductive synthesis technique [1, Examples 5–11] were successfully verified in KeYmaera X. The proof for the largest, 6-dim. nonlinear ODE [1, Example 5] required substantial manual arithmetic reasoning in KeYmaera X.[7]

The arithmetical conditions in [1, Equation 1] are identical to the premises of rule $\mathrm{Lyap}_{\geq}$ except it unsoundly omits the condition $v(0) = 0$, see supplement [35]. The generated Lyapunov functions remain correct because the inductive synthesis technique [1] implicitly guarantees this omitted condition.

*Summary.* These case studies demonstrate the feasibility of carrying out proofs of various (advanced) stability properties within KeYmaera X using this paper's stability specifications. The proofs share similar high-level proof structure, which suggests that proof automation could significantly reduce proof effort [10]. Such automation should also support user input of key insights for difficult reasoning steps, e.g., real arithmetic reasoning with nested, alternating quantifiers.

[7] The Lyapunov function in [1, Example 5] does *not* work for its associated ODE. It works if the ODE is corrected with $\dot{x}_1 = -x_1^3 + 4x_2^3 - 6x_3x_4$, as in the literature [23].

### 6 Related Work

Stability is a fundamental property of interest across many different fields of mathematics [6,15,19,30,31,34] and engineering [14,18,20]. This related work discussion focuses on formal approaches to stability of ODEs.

*Logical specification of stability.* Rouche, Habets, and Laloy [31] provide a pioneering example of using logical notation to specify and classify stability properties of ODEs. Alternative logical frameworks have also been used to specify stability and related properties: stability is expressed in HyperSTL [22] as a hyperproperty relating the trace of an ODE against two constant traces; $\epsilon$-stability is studied in the context of $\delta$-complete reasoning over the reals [12]; region stability for hybrid systems [29] is discussed using CTL*; the syntactic specification of $\mathrm{Asym}(x' = f(x), P)$ resembles the limit definition using filters [16]. This paper uses dL as a *sweet spot* logical framework, general enough to specify various stability properties of interest, e.g., asymptotic or exponential stability, and the stability of sets, while also enabling syntactic proofs of those properties.

*Formal verification of stability.* There is a vast literature on finding Lyapunov functions for stability, e.g., through numerical [24,23,37] and algebraic methods [9,21]. Formal approaches are often based on finding Lyapunov function candidates and *certifying* the correctness of those generated candidates [1,12,17,33]. This paper's approach enables highly trustworthy certification of those candidates in dL and KeYmaera X, with stability proof rules that are soundly *derived* from dL's parsimonious axiomatization [25,26,27], as implemented in KeYmaera X [11,26]. Sections 4 and 5 further show that this paper's approach supports verification of advanced stability properties [12,14,18] within the same dL framework. New stability proof rules like GLyap can also be soundly and *syntactically* justified in dL without the need for (low-level) semantic reasoning about the underlying ODE mathematics. As an example of the latter, semantic approach, LaSalle's invariance principle is formalized in Coq [7] and used to verify the correctness of an inverted pendulum controller [32].

### 7 Conclusion

This paper shows how ODE stability can be formalized in dL using the key idea that stability properties are $\forall/\exists$-quantified dynamical formulas. These specifications, their proof rules, and their logical relationships are all syntactically derived from dL's sound proof calculus. This further enables trustworthy KeYmaera X proofs that rigorously verify *every step* in an ODE stability argument, from arithmetical premises down to dynamical reasoning for ODEs. Directions for future work include *i)* formalization of stability with respect to perturbations of the system dynamics, and *ii)* generalizations of stability to hybrid systems.

**Acknowledgments.** We thank Brandon Bohrer, Stefan Mitsch, and the anonymous reviewers for their helpful feedback on KeYmaera X and this paper.
